# Enzymatic Characterization of Insecticide Resistance Mechanisms in Field Populations of Malaysian *Culex quinquefasciatus* Say (Diptera: Culicidae)

**DOI:** 10.1371/journal.pone.0079928

**Published:** 2013-11-21

**Authors:** Van Lun Low, Chee Dhang Chen, Han Lim Lee, Tiong Kai Tan, Chin Fong Chen, Cherng Shii Leong, Yvonne Ai Lian Lim, Phaik Eem Lim, Yusoff Norma-Rashid, Mohd Sofian-Azirun

**Affiliations:** 1 Institute of Biological Sciences, Faculty of Science, University of Malaya, Kuala Lumpur, Malaysia; 2 Medical Entomology Unit, WHO Collaborating Centre for Vectors, Institute for Medical Research, Kuala Lumpur, Malaysia; 3 Department of Parasitology, Faculty of Medicine, University of Malaya, Kuala Lumpur, Malaysia; 4 Institute of Ocean and Earth Sciences, University of Malaya, Kuala Lumpur, Malaysia; Instituto de Biotecnología, Universidad Nacional Autónoma de México, Mexico

## Abstract

**Background:**

There has been no comprehensive study on biochemical characterization of insecticide resistance mechanisms in field populations of Malaysian *Culex quinquefasciatus*. To fill this void in the literature, a nationwide investigation was performed to quantify the enzyme activities, thereby attempting to characterize the potential resistance mechanisms in *Cx. quinquefasciatus* in residential areas in Malaysia.

**Methodology/Principal Findings:**

*Culex quinquefasciatus* from 14 residential areas across 13 states and one federal territory were subjected to esterases, mixed function oxidases, glutathione-S-transferase and insensitive acetylcholinesterase assays. Enzyme assays revealed that α-esterases and β-esterases were elevated in 13 populations and 12 populations, respectively. Nine populations demonstrated elevated levels of mixed function oxidases and glutathione-S-transferase. Acetylcholinesterase was insensitive to propoxur in all 14 populations. Activity of α-esterases associated with malathion resistance was found in the present study. In addition, an association between the activity of α-esterases and β-esterases was also demonstrated.

**Conclusions/Significance:**

The present study has characterized the potential biochemical mechanisms in contributing towards insecticide resistance in *Cx. quinquefasciatus* field populations in Malaysia. Identification of mechanisms underlying the insecticide resistance will be beneficial in developing effective mosquito control programs in Malaysia.

## Introduction

Insecticide resistance mechanisms have been the subject of research interest among researchers from different parts of the world, including Malaysia. It has been proven that increased levels of mixed function oxidases contribute resistance to four major insecticide classes (i.e., organochlorines, carbamates, organophosphates and pyrethroids) [1]–[3]. It has also been reported that elevated levels of esterases are responsible for the resistance to organophosphates, carbamates and pyrethroids [4]–[5]. Involvement of glutathione-S-transferase in resistance to organophosphates, organochlorines and pyrethroids has also been noted [6]–[8]. Furthermore, previous studies have provided evidence on the role of insensitive acetylcholinesterase in resistance to organophosphates and carbamates [9]–[10]. As far as insecticide resistance mechanisms are concerned in Malaysia, a considerable amount of research indicated that Malaysian mosquitoes have demonstrated variable biochemical mechanisms in resistance to various insecticide classes [11]–[25].

With regard to *Culex quinquefasciatus* Say, it has been deemed as one of the three ‘world’s resistant mosquitoes’ [26]. The first documented case of insecticide resistance (towards organochlorines) in this mosquito species had been reported in 1952 in California [27]. Subsequently, the widespread development of its biotypes with resistance to 35 insecticide active ingredients has been documented worldwide [26]. In particular, Malaysian *Cx. quinquefasciatus*, the most abundant and annoying mosquito [28]–[29] has developed resistance towards four major insecticide classes [30]–[34].

Enzyme assay has been commonly used due to its rapid, simple and sensitive method for the identification of mechanisms underlying the insecticide resistance in mosquito population even at low frequencies [11], [35]. However, in Malaysia, the characterization of biochemical mechanisms of *Cx. quinquefasciatus* has been restricted to the districts of Kuala Lumpur [11]–[14], [16], [25], Sarawak [18] and laboratory insecticide selected strains [15]–[16], [19], [25]. Indeed, there has been a lack of evidence regarding the underlying mechanisms that are involved in insecticide resistance in the field populations of *Cx. quinquefasciatus* from other districts in Malaysia. Although previous studies have investigated certain enzymes in insecticide resistance development, there have been no comprehensive studies which concurrently characterize the α-esterases, β-esterases, mixed function oxidases, glutathione-S-transferase and insensitive acetylcholinesterase in resistance to four major insecticide classes. It is of great concern that the biochemical mechanisms in Malaysian *Cx. quinquefasciatus* populations could be underestimated, especially when there is an occurrence of multiple-resistant isolates within the same population.

Recently, multiple resistance to a broad spectrum of insecticides (i.e., DDT, propoxur, malathion and permethrin) ranged from susceptible, low to high resistance has been reported in Malaysian *Cx. quinquefasciatus* [34]. However, the actual mechanism(s) that conferred the development of insecticide resistance in these populations remain uncharacterized. In this context, a nationwide investigation was further conducted to (1) quantify the enzyme activities in field populations of *Cx. quinquefasciatus*, as part of an ongoing insecticide resistance monitoring from 14 residential areas across 11 states and one federal territory in Peninsular Malaysia and two states in East Malaysia, and thereby attempting to (2) correlate the degree of insecticide resistance with the levels of enzyme activities in this mosquito species. The present study is the first attempt to investigate the potential resistance mechanisms involving α-esterases, β-esterases, mixed function oxidases, glutathione-S-transferase and insensitive acetylcholinesterase towards resistance to DDT, propoxur, malathion and permethrin in *Cx. quinquefasciatus* from all states in Malaysia. With the continued use of insecticides, a better understanding of the prevailing insecticide resistance mechanisms could serve as a justification for changes in control practices and provide baseline data for population monitoring in accordance with appropriate insecticides.

## Materials and Methods

### Ethical Notes

This research was regulated by the Medical Review & Ethics Committee (MREC), Ministry of Health Malaysia. No specific permits were required for this study which did not involve endangered or protected species. Permission for the study to be conducted on private land/private residences was obtained from owners/residents prior to specimen collection.

### Mosquito Strains

**Figure 1:**
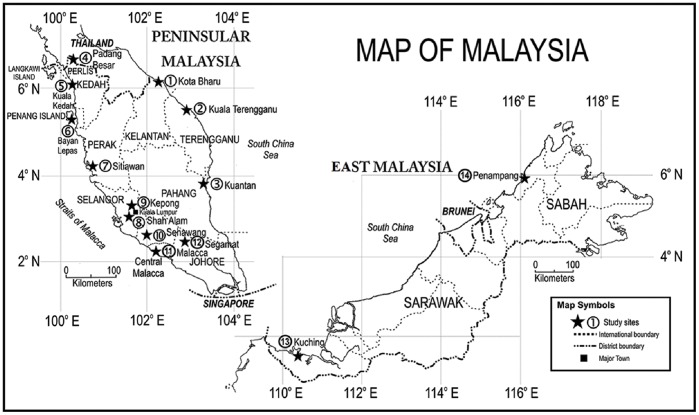
Collection sites of *Culex quinquefasciatus* across all states in Malaysia. *Figure reproduced with permission [34].

Given that there is no specific *Culex* control program in Malaysia, the study sites were selected on the basis of the incidence of dengue infestations and fogging activities, as intense fogging would inadvertently contaminate the breeding ground and exert selective pressure on *Cx quinquefasciatus*. A standardized larval dipping method developed by Mendoza *et al*. [36] was conducted. Mosquito larvae were collected from stagnant water in 14 residential areas across 13 states and a federal territory (i.e., Kuala Lumpur) in Malaysia (Figure 1). Details of the studied areas have been described elsewhere [34]. Field-collected larvae were transported to the Laboratory of Zoological and Ecological Research Network, University of Malaya and reared to adulthood for identification, using the taxonomic keys by Rattanarithikul *et al*. [37]. The identified *Cx. quinquefasciatus* female mosquitoes were blood-fed for the production of the first generation (F1). The non-blood fed three to five days old female mosquitoes from F1 reared larvae were used for WHO insecticide susceptibility tests (details have been published in previous report [34]) and biochemical assays. For comparison purposes, a laboratory reference strain of *Cx. quinquefasciatus* from the Institute for Medical Research, Kuala Lumpur, which has been cultured under insecticide free conditions for 117 generations was used. All female mosquitoes which have not been exposed to any chemicals were stored in −80°C freezer prior to the biochemical tests. In the present study, a total of 1,440 adult *Cx. quinquefasciatus* with 24 individual mosquitoes representing each of the 60 strains (four enzyme assays in 15 populations, including laboratory reference strain) were used.

### Enzyme Assays

Non-specific esterases enzyme assay was carried out according to established protocols [11], [38]. A total of 24 individual mosquitoes were homogenized in phosphate buffer solution and were centrifuged at 15,000 rpm for 10 minutes at 4°C. Four replicates of homogenate (50 µl) from each individual mosquito were obtained in this assay. The 50 µl of substrate solution (either α-naphthyl acetate or β-naphthyl acetate) was placed in a 96 well plate and left to stand for one minute, followed by the addition of 50 µl of 3 mM indicator solution (fast blue B salt). The reaction was further incubated for 10 minutes and was stopped by the addition of 50 µl of 10% acetic acid. The optical density was measured at 450 nm using absorbance microplate reader (BIO-TEK® ELx800™).

Mixed function oxidases enzyme assay was performed according to the method described by Brogdon *et al*. [39]. A total of 24 individual mosquitoes were homogenized in sodium acetate buffer solution and four replicates of homogenate (100 µl) from each individual mosquito were obtained in this assay. The optical density was measured at 630 nm after five minutes incubation of individual mosquito homogenate in each well with 200 µl of 2 mM 3,3′5,5′-tetramethylbenzidine (TMBZ) and 25 µl of 3% hydrogen peroxide.

Glutathione-S-transferase enzyme assay was conducted according to previously described protocol [15]. A total of 24 individual mosquitoes were homogenized in potassium phosphate buffer solution and were centrifuged at 14,000 rpm for 10 minutes at 4°C. Four replicates of homogenate (100 µl) from each individual mosquito were placed in a 96 well plate, followed by the addition of 50 µl of 2 mM glutathione and 50 µl of 1 mM 1-chloro-2, 4-dinitrobenzene (CDNB). The reaction was further incubated for 30 minutes, followed by the measurement of optical density at 400 nm.

With regard to insensitive acetylcholinesterase, enzyme assay was performed according to the method of Brogdon *et al*. [38], with minor modifications. In this assay, a total of eight replicates of homogenate (50 µl) from each individual mosquito were obtained. Briefly, the first batch of 12 individual mosquitoes which filled in the 96 wells was homogenized in potassium phosphate buffer and was centrifuged at 14,000 rpm for 10 minutes at 4°C. A 50 µl of reaction mixture containing 10% acetone buffer solution of 2.6 mM acetylthiocholine iodide (ACTHI), 0.3 mM of 5, 5-dithiobis (2-nitrobenzoic acid) (DTNB) and 0.1% propoxur inhibitor were added into each well. As for positive control, a 50 µl of reaction mixture without inhibitor was designed. The reaction was incubated at room temperature (28°C) for 30 minutes, followed by the measurement of optical density at 400 nm. This procedure was repeated for the second batch of 12 individual mosquitoes.

### Statistical Analysis

Spearman rank-order correlation using SPSS (ver 18) was performed to (1) determine the associations between the survivability rates in adult bioassays and enzyme activities, (2) investigate the relationships between enzyme activities.

Comparative measure of mean enzyme activities between the study sites was performed by one-way analysis of variance (ANOVA) using SPSS (ver 18). Tukey’s test was used to separate means in significant ANOVAs, *P*<0.05. Independent-samples t-test was performed to indicate significant increase in mean differences.

With respect to insensitive acetylcholinesterase, results were interpreted as a percentage remaining activity in the propoxur inhibited fraction compared to the control (uninhibited) activity [40]. Individual mosquitoes with more than 70% remaining activity are indicative of homozygous resistance (RR), 30–70% remaining activity are indicative of heterozygous (RS) and less than 30% remaining activity are indicative of homozygous susceptible (SS). Because of the light absorbance of propoxur in the microplate, in certain cases, homogenates appear to show higher acetylcholinesterase activity in propoxur-inhibited fraction (>100%) and it is normal in resistant strains [40].

## Results

WHO adult bioassays demonstrated a broad spectrum of susceptibility status against DDT, propoxur, malathion and permethrin across all study sites [34]. Adult mortality recorded 24 h after the initial exposure period of DDT, propoxur, malathion and permethrin ranged from 0.00 to 40.00, 3.34 to 68.89, 0.00 to 100.00 and 36.67 to 100.00%, respectively. Generally, DDT and propoxur resistance were expressed most frequently, as all study sites demonstrated a resistant biotype (less than 80% mortality). With regard to malathion and permethrin, a resistant biotype was detected from 11 out of 14 and 6 out of 14 of the populations, respectively (Table 1).

**Table 1:** Mortality of Malaysian *Culex quinquefasciatus* adults using a WHOPES treated filter paper assay.

Strain	DDT (4.0%)	Propoxur (0.1%)	Malathion (5.0%)	Permethrin (0.25%)
Reference	43.34±2.72	100.00±0.00	100±0.00	100.00±0.00
Kelantan	^R^24.44±4.44	^R^3.34±3.34	^M^96.67±3.34	^R^43.33±10.00
Terengganu	^R^25.00±5.00	^R^55.00±5.00	^S^100.00±0.00	^M^95.00±5.00
Pahang	^R^13.33±3.33	^R^20.00±5.77	^S^100.00±0.00	^R^36.67±3.33
Perlis	^R^2.22±2.22	^R^68.89±5.88	^R^75.55±2.22	^R^71.11±2.22
Kedah	^R^4.45±2.22	^R^6.67±3.85	^R^71.11±5.88	^R^37.78±2.22
Penang	^R^35.56±5.88	^R^37.78±2.22	^R^35.56±5.88	^M^76.67±10.00
Perak	^R^20.00±0.00	^R^6.67±6.67	^R^10.00±3.33	^M^83.33±10.00
Selangor	^R^6.67±3.85	^R^55.55±2.22	^R^0.00±0.00	^R^62.22±4.45
Kuala Lumpur	^R^17.78±2.22	^R^33.33±3.85	^R^11.11±5.88	^R^77.78±2.22
Negeri Sembilan	^R^20.00±0.00	^R^10.00±5.77	^R^6.67±6.67	^S^100.00±0.00
Malacca	^R^11.11±2.22	^R^15.55±2.22	^R^4.44±4.44	^M^82.22±5.88
Johore	^R^2.22±2.22	^R^22.22±2.22	^R^31.11±4.44	^S^100.00±0.00
Sarawak	^R^0.00±0.00	^R^53.33±3.85	^R^62.22±5.88	^S^100.00±0.00
Sabah	^R^40.00±3.85	^R^62.22±4.45	^R^55.55±2.22	^M^93.33±0.00

Details have been produced in previous study [34].

R = resistant, S = susceptible, M = moderate resistant as determined by WHO [50].

**Figure 2:**
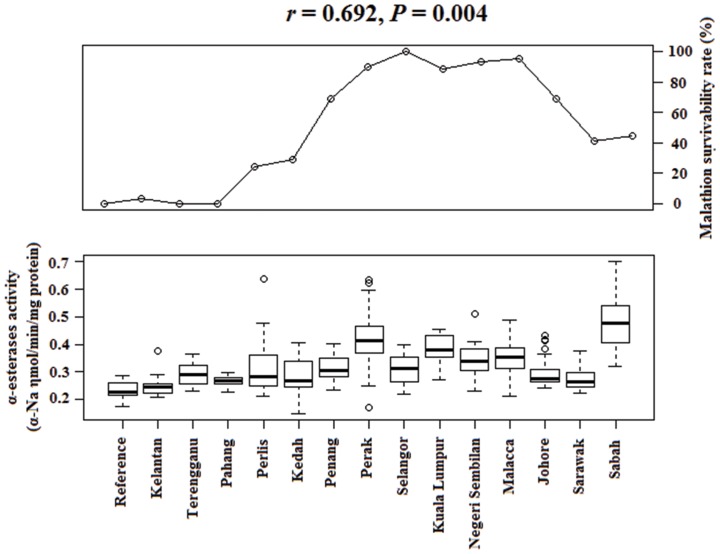
Spearman rank-order correlation between malathion survivability rate and α-esterases activity in Malaysian *Culex quinquefasciatus*.

**Figure 3:**
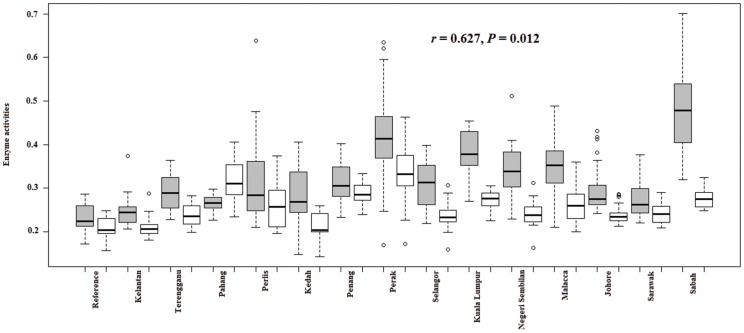
Spearman rank-order correlation between the activity of α-esterases (grey) and β-esterases (white) in Malaysian *Culex quinquefasciatus*.

One-way ANOVA revealed that the mean of all tested enzyme activities in Malaysian *Cx. quinquefasciatus* were significantly different across all study sites (*P*<0.001). In addition, Spearman rank-order correlation indicated a significant correlation between malathion survivability rate in adult bioassays and α-esterases activity in Malaysian *Culex quinquefasciatus* (*r* = 0.692, *P* = 0.004) (Figure 2), while no correlation was found with other insecticide survivability rates against others enzyme activities. An association between the activity of α-esterases and β-esterases (*r* = 0.627, *P* = 0.012) was also demonstrated (Figure 3).

In non-specific esterases assay, a significant increase in α-esterases activity was detected in all populations (except Kelantan). A lack of elevated β-esterases activity was observed in Kelantan and Kedah populations, whereas other populations exhibited a significant increase in β-esterases activity. All populations exhibited higher α-esterases activity, as compared to β-esterases activity (except Pahang) (Table 2).

**Table 2:** Mean (± SE) esterases, glutathione-S-transferases and mixed function oxidases and acetylcholinesterase activities in Malaysian *Cx. quinquefasciatus* populations.

	α-EST	β-EST	MFO	GST	AChE	pAChE
Strain	(α-Na ηmol/min/mg protein)	(β-Na ηmol/min/mg protein)	(Absorbance 630 nm)	(CDNA-ηmol/min/mg protein)	(Control withoutinsecticide)	(ACTH with 0.1%propoxur)
Reference	0.23±0.01	0.21±0.00	0.50±0.02	0.13±0.00	0.17±0.01	0.05±0.00
Kelantan	0.25±0.01^a^	0.21±0.00^a^	0.53±0.01^a^	*0.15±0.00^def^	*0.22±0.01^bcd^	*0.09±0.00^a^
Terengganu	*0.29±0.01^abc^	*0.24±0.01^abc^	*1.08±0.05^e^	*0.15±0.00^def^	*0.32±0.01^fg^	*0.09±0.00^ab^
Pahang	*0.27±0.00^ab^	*0.32±0.01^ef^	0.42±0.02^a^	*0.16±0.00^ef^	*0.24±0.01^cde^	*0.09±0.00^ab^
Perlis	*0.32±0.02^bc^	*0.26±0.01^bcd^	0.53±0.02^a^	0.13±0.01^abc^	0.16±0.01^ab^	*0.09±0.00^ab^
Kedah	*0.29±0.01^abc^	0.21±0.01^a^	*0.94±0.05^de^	*0.16±0.01^f^	*0.35±0.02^g^	*0.10±0.00^ab^
Penang	*0.32±0.01^bc^	*0.29±0.00^de^	*0.93±0.06^d^	*0.17±0.00^f^	*0.28±0.01^def^	*0.12±0.00^c^
Perak	*0.42±0.02^ef^	*0.34±0.01^f^	*0.57±0.02^ab^	*0.15±0.00^def^	*0.29±0.02^efg^	*0.09±0.00^ab^
Selangor	*0.31±0.01^bc^	*0.24±0.01^ab^	*0.83±0.02^cd^	0.12±0.00^a^	*0.24±0.01^cde^	*0.10±0.00^ab^
Kuala Lumpur	*0.38±0.01^de^	*0.27±0.00^cd^	0.44±0.01^a^	0.12±0.00^a^	0.16±0.01^ab^	*0.09±0.00^a^
Negeri Sembilan	*0.34±0.01^cd^	*0.24±0.01^abc^	*0.84±0.03^d^	0.14±0.00^bcd^	*0.24±0.02^cde^	*0.10±0.00^b^
Malacca	*0.34±0.01^cd^	*0.26±0.01^bcd^	*0.96±0.02^de^	*0.14±0.00^cde^	*0.21±0.01^bc^	*0.10±0.00^ab^
Johore	*0.30±0.01^abc^	*0.24±0.00^abc^	0.53±0.02^ab^	0.12±0.00^ab^	*0.20±0.01^bc^	*0.09±0.00^ab^
Sarawak	*0.28±0.01^ab^	*0.24±0.00^abc^	*0.87±0.04^d^	*0.14±0.00^cde^	0.12±0.00^a^	*0.09±0.00^ab^
Sabah	*0.47±0.02^f^	*0.28±0.00^d^	*0.68±0.03^bc^	*0.14±0.00^cde^	*0.21±0.01^bc^	*0.10±0.00^b^
One way ANOVA	*F* = 21.43; df = 13,322; *P*<0.0001	*F = *25.99; df = 13,322; *P*<0.0001	*F* = 46.98; df = 13,322; *P*<0.0001	*F* = 18.75; df = 13,322; *P*<0.0001	*F = *22.25; df = 13,322; *P*<0.0001	*F* = 7.65; df = 13,322; *P*<0.0001

α-EST = α-esterases, β-EST = β-esterases, MFO = mixed function oxidases, GST = glutathione-S-transferase, AChE = acetylcholinesterase, pAChE = propoxur-inhibited acetylcholinesterase. Mean followed by a different letter were significantly different, *P*<0.05, Tukey’s test.

* Significant increase in mean differences compared to the laboratory reference strain, *P*<0.05, t-test.

As for mixed function oxidases assay, an elevated level of mixed function oxidases activity was found in nine populations (i.e., Kedah, Malacca, Negeri Sembilan, Penang, Perak, Sabah, Selangor, Sarawak and Terengganu) (Table 2).

Of 14 populations, nine populations (i.e., Kedah, Kelantan, Malacca, Pahang, Penang, Perak, Sabah, Sarawak and Terengganu) exhibited a significant increase in glutathione-S-transferase activity (Table 2).

**Figure 4:**
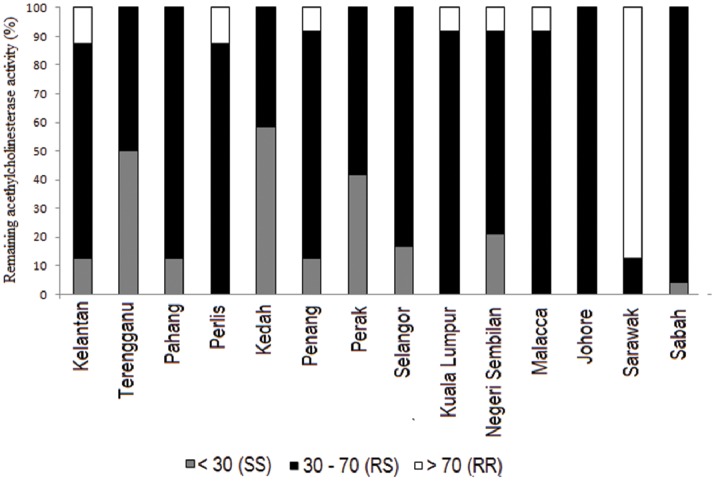
Percentage remaining activity of acetylcholinesterase in individual Malaysian *Culex quinquefasciatus* after 0.1% propoxur inhibition. *<30% = homozygous susceptible (SS), 30–70% = heterozygous (RS), >70% = homozygous resistance (RR) [40].

With regard to insensitive acetylcholinesterase assay, all populations revealed a significant increase in acetylcholinesterase activity in the control test (absence of propoxur), except Kuala Lumpur, Perlis and Sarawak. In comparison to the laboratory strain, all populations also revealed a significant increase in acetylcholinesterase activity in the presence of propoxur (Table 2). A quick perusal of the remaining activity data indicated that RS was detected in all 14 populations. The RS genotype was also the most prevalent, with 246 individuals from a total sample size of 336, followed by SS genotype (55 individuals) and RR genotype (35 individuals). An excess of RR genotype was recorded in *Cx. quinquefasciatus* population from Sarawak (Figure 4).

Summary of insecticide resistance and prevalence of resistance mechanisms in different *Cx. quinquefasciatus* populations was presented in Table 3. Elevated levels of all enzymes activities were demonstrated in four populations (Malacca, Penang, Perak and Terengganu).

**Table 3:** Summary of insecticide resistance and prevalence of resistance mechanisms in different *Cx. quinquefasciatus* populations in Malaysia.

	Insecticide resistance				Elevated enzyme activity					
Strain	DDT	PRO	MAL	PER	α-EST	β-EST	MFO	GST	AChE	pAChE
Kelantan	R	R	M	R	−	−	−	+	+	+
Terengganu	R	R	S	M	+	+	+	+	+	+
Pahang	R	R	S	R	+	+	−	+	+	+
Perlis	R	R	R	R	+	+	−	−	−	+
Kedah	R	R	R	R	+	−	+	+	+	+
Penang	R	R	R	M	+	+	+	+	+	+
Perak	R	R	R	M	+	+	+	+	+	+
Selangor	R	R	R	R	+	+	+	−	+	+
Kuala Lumpur	R	R	R	R	+	+	−	−	−	+
Negeri Sembilan	R	R	R	S	+	+	+	−	+	+
Malacca	R	R	R	M	+	+	+	+	+	+
Johore	R	R	R	S	+	+	−	+	+	+
Sarawak	R	R	R	S	+	+	+	+	−	+
Sabah	R	R	R	M	+	+	+	−	+	+

* PRO = propoxur, MAL = malathion, PER = permethrin, α-EST = α-esterases, β-EST = β-esterases, MFO = mixed function oxidases, GST = glutathione-S-transferase, AChE = acetylcholinesterase, pAChE = propoxur-inhibited acetylcholinesterase, R = resistant, M = moderate resistant, S = susceptible, + = presence of mechanism, − = absence of mechanism.

## Discussion

Regardless of the associations between the degree of insecticide resistance and the enzyme activities, the enhanced enzyme activities of α-esterases, β-esterases, mixed function oxidases, glutathione-S-transferase and insensitive acetylcholinesterase in Malaysian *Cx. quinquefasciatus* confirmed the incidence of insecticide resistance (low to high resistance towards DDT, propoxur, malathion and permethrin), as detected by WHO adult bioassays [34]. An elevated level of esterases [11]–[13], [16], oxidases [17]–[18], [25] in field populations of Malaysian *Cx. quinquefasciatus* has been previously described. However, the elevated levels of glutathione-S-transferase and acetylcholinesterase in this study indicated contrasting results with previous studies, where there was a lack of elevated level of glutathione-S-transferase and acetylcholinesterase after propoxur inhibition in Malaysian *Cx. quinquefasciatus* [15], [18]. The appearance of these switching patterns of resistance mechanisms has provided an emergency notification to local authorities concerning the evolution of multiple insecticide resistance in this problematic pest species. The elevated levels of enzyme activities are indeed placing an increasing burden on current and future mosquito control practices in the country. The present findings highlight the urgent need for appropriate resistance management strategies to be implemented in vector control programs in Malaysia.

Statistical analysis demonstrated that there was a significant association between malathion survivability rate in adult bioassays and α-esterases activity in Malaysian *Culex quinquefasciatus*, suggested that the development of malathion resistance in these populations was due to the increased levels of α-esterases activity. Elevated esterases levels associated with organophosphate resistance in Malaysian *Cx. quinquefasciatus* has also been reported in earlier studies [11]–[12]. Likewise, similar detoxification mechanism has also been found in Malaysian *Aedes aegypti* [20]. Nonetheless, little attention has been paid to the comparative study of both α-esterases and β-esterases in Malaysian mosquitoes. It is worth mentioning that the α-esterases in Malaysian mosquitoes have been the focus of study while β-esterases have been totally disregarded. The comparative study performed in the present study indicated that activity of α-esterases was higher than β-esterases in all populations (except Pahang). Similar observation has also been found in *Ae. aegypti* and *Ae. albopictus* populations in Thailand [10]. Meanwhile, inconsistent trends in both α-esterases and β-esterases activities have been demonstrated in Indian *Cx. quinquefasciatus* populations [41].

With regard to other insecticide survivability rates against any other enzyme activities, no correlation was found in the present study. However, previous studies reported that elevated levels of oxidases were correlated with pyrethroid resistance in Malaysian *Cx. quinquefasciatus* [25], *Ae. albopictus* [22], [24], and *Ae. Aegypti* [23]. Although glutathione-S-transferase is responsible for resistance to various insecticide classes across a number of insect species, it has primarily been associated with DDT resistance [42]. Earlier study has attempted to establish the correlation between glutathione-S-transferase activity and DDT resistance in Malaysian *Anopheles maculatus*, *Cx. quinquefasciatus* and *Ae. aegypti*, but failed to demonstrate a clear relationship [15]. Even today, the associations between glutathione-S-transferase activity and insecticide resistance in Malaysian mosquitoes were poorly evidenced. Prior to 1964, DDT was used in a pilot Malaria Eradication Programme (MEP) in Negeri Sembilan at the standard dosage of 2g a.i./m^2^. Thereafter, DDT was officially introduced in 1967 as an indoor residual spray when the MEP was officially launched. However, its use was stopped in 1998 and replaced by permethrin and deltamethrin until today. It is important to emphasize that DDT has not been applied in Malaysia since 1998, but the resistance phenotype still remains in Malaysian populations [34]. It certainly does not exclude the consequence of the extensive use of pyrethroids in recent years which also conferred resistance to DDT, as both pyrethroids and DDT are specifically designed to attack the voltage-gated sodium channel of insect [43]. One plausible explanation for the lack of association between DDT resistance and glutathione-S-transferase activity could be the evolution of knockdown resistance. The widespread distribution of L1014F mutation across the country has been reported recently in this species [44]. Therefore, it is speculated that DDT resistance evolved in Malaysian *Cx. quinquefasciatus* was due to the joint action of knockdown resistance and detoxification mechanisms.

As for insensitive acetylcholinesterase, a retrospective study reported that the acetylcholinesterase activity in Malaysian *Cx. quinquefasciatus* has been sensitive to propoxur [12]. However, the present results have provided strong evidence on the role of insensitive acetylcholinesterase in the development of propoxur resistance in Malaysian *Cx. quinquefasciatus*. Propoxur has never been used in government vector control programs in Malaysia. It was introduced in the early 1970’s as household aerosol and widely used by house owners to control pests including mosquitoes. Its use in aerosol form was stopped in the 1990s. In point of fact that this species prefers to rest indoor, it was more likely to be exposed to household aerosol containing propoxur as the active ingredient, thereby contributing to the development of propoxur resistance. In conjunction with propoxur-insensitive acetylcholinesterase, the first molecular genotyping of insensitive acetylcholinesterase associated with malathion resistance in Malaysian *Cx. quinquefasciatus* has also been documented [45]. With the information presented in this study, cross-resistance between propoxur and malathion due to insensitive acetylcholinesterase was conclusively identified. Cross-resistance was similarly detected in previous study [34].

Multiple insecticide resistance implied that more than one mechanism is involved in insecticide resistance. In fact, the evolution of multiple-resistant isolates is not a new phenomenon and is becoming problematic worldwide. In the present study, an association between activity of α-esterases and β-esterases was demonstrated. The association between the activity of α-esterases and β-esterases has also been documented previously [46] and it is proposed that the occurrence of this incidence might be due to the co-amplification of two esterase genes (estα2^1^ and estβ2^1^) which was commonly found in organophosphate-resistant *Cx. quinquefasciatus* [43].

In conclusion, the results presented here provide the first report on the mechanisms of α-esterases, β-esterases, mixed function oxidases, glutathione-S-transferase and acetylcholinesterase towards resistance to DDT, propoxur, malathion and permethrin in *Cx. quinquefasciatus* from all states in Malaysia. Evidence of malathion resistance due to elevated α-esterases activity was found. In addition, an association between activity of α-esterases and β-esterases was also demonstrated in the present study. Nevertheless, multiple insecticide resistance involving both metabolic mechanisms and target site alteration in *Cx. quinquefasciatus* has been reported from many parts of the world [41], [47]–[48]. Furthermore, reduced insecticide penetration (cuticular resistance) in *Cx. quinquefasciatus* has also been noted [49]. Hence, for future study, investigation of the insecticide resistance involving the factors mentioned above in Malaysian *Cx. quinquefasciatus* will be beneficial in unraveling the prevailing resistance mechanisms which will therefore contribute to the technical know-how of implementing effective mosquito control programs in Malaysia.
